# Parental smoking and blood pressure in children and adolescents: a national cross-sectional study in China

**DOI:** 10.1186/s12887-019-1505-8

**Published:** 2019-04-18

**Authors:** Zilong Zhang, Jun Ma, Zhenghe Wang, Yanhui Dong, Zhaogeng Yang, Bin Dong, Yinghua Ma

**Affiliations:** 1 Institute of Child and Adolescent Health, School of Public Health, Peking University Health Science Center, Xueyuan Road 38, Haidian District, Beijing, 100191 China; 2 Public Health Ontario, Toronto, ON Canada

**Keywords:** Parental smoking, Children and adolescents, Blood pressure, Hypertension

## Abstract

**Background:**

Current evidence on the health effects of passive smoking on childhood blood pressure is limited and inconsistent. We investigated the associations between exposure to parental smoking and blood pressure in children and adolescents.

**Methods:**

A cross-sectional analysis was performed in a national sample of 42,745 children and adolescents (50.2% boys) aged 7–18 years from seven provinces in China. Systolic blood pressure (SBP) and diastolic blood pressure (DBP) were measured. Information on parental smoking was collected through questionnaire. Multivariable linear regression and logistic regression was used to investigate the associations of parental smoking with blood pressure and prevalent hypertension, respectively.

**Results:**

The reported parental smoking rates were 49.7 and 50.2% in boys and girls, respectively. After adjustment for a range of potential confounders, exposure to parental smoking was associated with 0.44 [95% confidence interval (CI): 0.16, 0.72] mmHg and 0.26 (95% CI: 0.04, 0.47) mmHg higher SBP and DBP in girls. Girls exposed to parental smoking were also more likely to have hypertension compared with those without exposure (odds ratio = 1.11, 95% CI: 1.02, 1.20). No significant associations were found in boys.

**Conclusions:**

Exposure to parental smoking was associated with increased blood pressure and higher prevalence of hypertension in girls, but not in boys. Urgent strategies are needed for the promotion of smoking-free environment, especially for children and adolescents.

**Electronic supplementary material:**

The online version of this article (10.1186/s12887-019-1505-8) contains supplementary material, which is available to authorized users.

## Background

Hypertension is a well-established risk factor for atherosclerosis, which is the underlying pathology of most cardiovascular diseases [1]. There is evidence showing that blood pressure tracks well from childhood to adulthood and elevated blood pressure in childhood is a good predictor of adult hypertension [2]. Consistent associations have also been shown between elevated blood pressure in childhood and impaired cardiovascular function in adulthood [3]. The blood pressure development in childhood and its tracking into adulthood is affected by various factors, including genetics, biology, behavior and social and environment factors [4–6].

Cigarette smoking is a major source of indoor air pollution. Side-stream cigarette smoke contains similar adverse components as mainstream cigarette smoke, and the concentrations of some chemicals may be even higher in side-stream smoke than in mainstream smoke [7]. Children are more vulnerable to the adverse effects of air toxicants, such as second-hand smoke, because of the underdeveloped lungs and greater exposure due to higher ventilation rate [8]. In recent years, a number of epidemiological studies investigated the associations between passive smoking, mainly due to parental smoking, and blood pressure in children and adolescents, but the results are inconsistent [9–17]. Moreover, the majority of these studies were conducted among Western populations and there is limited evidence in China. China is among the countries with highest prevalence of smoking and the smoking rate is especially high for Chinese men [18]. On the other hand, hypertension causes a substantial disease burden in China. In 2010, more than 2 million death were attributable to hypertension, accounting for 24.6% deaths in China [19]. Investigation on the health effects of parental smoking on childhood blood pressure may provide important evidence to aid the development of effective intervention strategies to control smoking and consequently to improve children’s cardiovascular health. We therefore assessed the associations between exposure to parental smoking and blood pressure and hypertension in a large population of Chinese children and adolescents.

## Methods

### Study participants

Study participants were from a national project in China which aimed to evaluate the effectiveness of a lifestyle intervention program on obesity prevention in children and adolescents. Details of the project have been documented elsewhere [20, 21]. In brief, more than 60,000 children and adolescents aged 7–18 years were recruited from seven provinces in China (Chongqing, Hunan, Guangdong, Liaoning, Ningxia, Shanghai and Tianjin) in 2013 through a multi-stage random sampling. Participants received a series of physical examinations, blood tests as well as a questionnaire investigation. The study protocol was approved by the Ethical Committee of Peking University and written informed consent was obtained from the participants and their guardians prior to their participation.

In the present study, we performed a cross-sectional analysis using baseline data of the project. Blood pressure measures were available for 599,59 children and adolescents. We excluded 16,732 participants with missing information on parental smoking and some other potential confounders (e.g. lifestyle and maternal educational level). We further excluded 482 participants who reported smoking behaviors to eliminate possible influences of active smoking. Finally, 42,745 participants (50.2% boys) were included in the data analysis.

### Data collection

Participants underwent a series of anthropometric measurements in the morning, including height, weight and blood pressure. Barefoot height was measured to the nearest 0.1 cm using a portable stadiometer (Model TZG, China). Weight was measured to the nearest 0.1 kg using a standardized scale with participants wearing light clothing. Body mass index (BMI) was calculated as weight (kg) divided by the square of height (m). Blood pressure was measured using an auscultation mercury sphygmomanometer (Model XJ1ID, China) with an appropriate cuff for children. Blood pressure measurement was performed after at least 5-min rest with participants in seated position. The cuff was placed around 2 cm above the crease of the right arm elbow. Systolic blood pressure (SBP) was determined by onset of the first Korotkoff sound (K1) and diastolic blood pressure (DBP) was determined by the fifth Korotkoff sound (K5). Blood pressure was measured twice with 1-min interval and children were asked to remain quiet and to sit still while each reading was being taken. The average of the two readings was calculated and then used in data analysis. All measurements were carried out by trained technicians. The equipment was calibrated regularly and the information on detailed procedures of quality control can be assessed in our published study protocol [20].

A self-administered questionnaire was used to collect information on participants` demographic information and lifestyle factors, such as exercise and diet. Information on parental smoking, parental education and family disease history was collected by a parental questionnaire. The smoking rate (current smoking) reported in the present study was much higher in fathers (49.7%) than in mothers (1.3%). Thus, we categorized parental smoking into two groups: non-exposure group (neither of the parent smoked) and exposure group (at least one parent smoked).

### Statistical analysis

Mean [standard deviation (SD)] and number (percentage) were used for descriptive statistics. Between-group comparisons were conducted using T test, chi-square test or Kruskal-Wallis rank test when appropriate. We used multivariable linear regression models to investigate the associations between exposure to parental smoking and blood pressure. We developed three models with potential confounders being added gradually, including: Crude Model: with no adjustment; Model 1: adjusted by age, height and BMI (all as continuous variables); Model 2: further adjusted by outdoor exercise (< 1 h/day, 1–2 h/day or ≥ 2 h/day), fruit intake (≤ 3 days/week, 4–6 days/week or daily), vegetable intake (≤ 3 days/week, 4–6 days/week or daily), maternal educational level (primary school or illiterate, secondary school, high school, or college/university or above) and parental hypertension (yes or no). All analyses were performed separately for boys and girls.

Logistic regression was used to examine the associations between parental smoking and prevalent hypertension. Hypertension was defined according to the national references for Chinese children and adolescents aged 7–17 years (SBP or DBP ≥ age, sex-and-height specific 95th percentiles) [21]. For participants aged 18 years, the cut-offs of 140/90 for adults were used.

We conducted a sensitivity analysis to control the possible influence of active smoking. We included the 482 participants who were previously excluded because of reported active smoking behaviors and introduced active smoking (yes or no) as a covariate into the regression models. We also performed a subgroup analysis by classifying the study participants into two groups: younger group (7–12 years of age) and older group (13–18 years of age). Active smoking is rare in younger children, and therefore findings from this group would be less likely to be affected by active smoking.

All statistical analyses were performed using R 3.4.3 (R Core Team, Vienna, Austria). A two-tailed *P* value of < 0.05 defined statistical significance.

## Results

General characteristics of the 21,463 boys and 21,282 girls included in data analysis are presented in Table 1. The mean age was 11.1 (SD: 3.1) years and 11.5 (SD: 3.2) years for boys and girls, respectively. Compared with girls, boys had greater values of all anthropometric measures. The prevalence of hypertension was also slightly higher in boys (13.6%) than in girls (12.8%). Boys were physically more active than girls. The distribution of parental smoking and maternal educational level was similar among boys and girls. The 42,745 participants included in data analysis were generally comparable with the whole study population in terms of the distribution of age, sex and anthropometric measures (Additional file 1).

**Table 1:** Characteristics of study participants

Characteristics	Boys (*N* = 21,463)	Girls (*N* = 21,282)	*P* for comparison
Age (year)	11.1(3.1)	11.5(3.2)	< 0.001
Weight (kg)	42.4(16.7)	39.8(13.6)	< 0.001
Height (cm)	146.7(17.6%)	145.1(14.8%)	< 0.001
Body mass index (kg/m^2^)	18.9(4.0)	18.3(3.6)	< 0.001
Systolic blood pressure (mmHg)	106.0(12.5)	103.3(11.6)	< 0.001
Diastolic blood pressure (mmHg)	66.8(8.8)	65.9(8.5)	< 0.001
Hypertension	2927(13.6%)	2727(12.8%)	0.01
Outdoor exercise			< 0.001
< 1 h/day	8333(38.8%)	9548(44.9%)	
1–2 h/day	8144(37.9%)	7629(35.8%)	
≥ 2 h/day	4986(23.2%)	4105(19.3%)	
Fruit intake			< 0.001
≤ 3 days/week	6431(30.0%)	5031(23.6%)	
4–6 days/week	6533(30.4%)	6770(31.8%)	
Daily	8944(39.9%)	9481(44.5%)	
Vegetable intake			< 0.001
≤ 3 days/week	2773(12.9%)	2485(11.7%)	
4–6 days/week	3515(16.4%)	3354(15.8%)	
Daily	15,175(70.7%)	15,443(72.6%)	
Parental smoking	10,668(49.7%)	10,684(50.2%)	0.30
Parental hypertension	1434(6.7%)	1565(7.4%)	0.007
Maternal educational attainment			0.08
Primary school or illiterate	2143(10.0%)	2068(9.7%)	
Secondary school	8173(38.1%)	7899(37.1%)	
High school	5534(25.8%)	5638(26.5%)	
College/university or above	5613(26.2%)	5677(26.7%)	

Descriptive statistics are presented as mean (SD) and number (percentage) for continuous variables and categorical variables, respectively

Table 2 shows the results of the associations between parental smoking and blood pressure from linear regression analysis. Positive associations between parental smoking and blood pressure were found in girls and the associations were stable after adjustment for a range of confounders. In fully adjusted model (Model 3), in comparison with girls without exposure to parental smoking, those in exposure group had 0.44 [95% confidence interval (CI): 0.16,0.72] mmHg and 0.26 (95% CI: 0.04,0.47) mmHg higher SBP and DBP, respectively. No significant associations between parental smoking and blood pressure were found in boys.

**Table 2:** Associations between exposure to parental smoking and blood pressure in children and adolescents

Blood Pressure	Boys (*N* = 21,463)		Girls (N = 21,282)	
	Coeff (95% CI)	*P*	Coeff (95% CI)	*P*
SBP (mmHg)				
Crude Model	0.30 (−0.03,0.64)	0.08	1.03 (0.72,1.34)	< 0.001
Model 1	0.24 (− 0.04,0.52)	0.09	0.68 (0.41,0.96)	< 0.001
Model 2	0.06 (−0.22,0.33)	0.69	0.44 (0.16,0.72)	0.002
DBP (mmHg)				
Crude Model	0.18 (−0.06,0.41)	0.14	0.60 (0.38,0.83)	< 0.001
Model 1	0.13 (−0.08,0.35)	0.23	0.40 (0.19,0.62)	< 0.001
Model 2	0.0001 (−0.21,0.21)	0.99	0.26 (0.04,0.47)	0.02

Participants reporting no exposure to parental smoking served as reference group

Crude Model: no adjustment; Model 1: adjusted for age, height and body mass index; Model 2: further adjusted for lifestyle factors (exercise, fruit intake and vegetable intake), maternal educational level and parental hypertension

Abbreviations: *CI* Confidence interval, *DBP* Diastolic blood pressure, *SBP* Systolic blood pressure

Consistent with the results of linear regression analysis, a positive association was detected between parental smoking and prevalent hypertension in girls. (Table 3)The girls with exposure to parental smoking had 1.11 higher odds of having hypertension than their counterparts without exposure [odds ratio (OR): 1.11, 95% CI: 1.02, 1.20]. Similarly, no significant associations were found in boys.

**Table 3:** Associations between exposure to parental smoking and hypertension in children and adolescents

Hypertension	Boys (N = 21,463)		Girls (*N* = 21,282)	
	OR (95% CI)	*P*	OR (95% CI)	*P*
Crude Model	1.01 (0.93,1.09)	0.90	1.19 (1.10,1.29)	< 0.001
Model 1	0.96 (0.89,1.04)	0.32	1.15 (1.06,1.25)	< 0.001
Model 2	0.93 (0.86,1.01)	0.09	1.11 (1.02,1.20)	0.01

Participants reporting no exposure to parental smoking served as reference group

Crude Model: no adjustment; Model 1: adjusted for age, height and body mass index; Model 2: further adjusted for lifestyle factors (exercise, fruit intake and vegetable intake), maternal educational level and parental hypertension

Abbreviations: *CI* Confidence interval, *DBP* Diastolic blood pressure, *OR* odds ratio, *SBP* Systolic blood pressure

In sensitivity analysis, the inclusion of participants with active smoking and additional adjustment for active smoking did not change the results materially (data not shown). In subgroup analysis, we found similar results in the younger group. Exposure to parental smoking was associated with increased blood pressure and higher prevalence of hypertension in girls, although the positive association between DBP and parental smoking did not reach statistical significance. No significant associations were found in younger boys. (Additional file 2).

## Discussion

To our knowledge, this is the largest epidemiological study to date which investigated the associations between exposure to parental smoking and blood pressure and hypertension in Chinese children and adolescent. We found that exposure to parental smoking was associated with increased SBP and DBP and higher prevalence of hypertension in girls. No significant associations were found in boys.

Current evidence on the associations between passive smoking, mainly parental smoking, and blood pressure of children and adolescents is inconsistent and controversial. In line with our findings, Schwandt and colleagues reported that parental smoking was associated with higher prevalence of hypertension in children in 2011 child-parent pairs in Germany [11]. In another study of 4236 German preschool children, both SBP (+ 1.0, 95% CI: + 0.5, + 1.5 mmHg) and DBP (+ 0.5, 95% CI: + 0.03, + 0.9 mmHg) were higher in children of smoking parents [13]. An Iranian study of 160 children also found that parental smoking was associated with increased SBP and DBP, but the significant associations diminished in sex-specific subgroup analysis [14]. In contrast, a number of other studies found no significant associations between childhood passive smoking and blood pressure in children and/or adolescents in Poland [9], Turkey [12], Belarus [15] and Italy [17]. Two other studies in the U.S. [10] and Iran [16] found that passive smoking was associated with higher prevalence of metabolic syndrome (MS), but not with elevated blood pressure, a single component of MS. More studies are needed to better investigate the health effects of parental smoking on blood pressure in children and adolescents, especially in regions with high smoking rate and great hypertension disease burden, such as China.

We are not sure about the underlying mechanisms responsible for the observed sex differences in the associations between parental smoking and blood pressure. One possible explanation is that girls were physically less active than boys and they might spend more time indoors (Table 1). Therefore, the exposure to passive smoking in girls could be greater. More future studies are warranted to elucidate the underlying mechanisms.

Our study has several important strengths. First, the large sample size along with the affluent information on a wide range of potential confounders enabled us to better characterize the associations between exposure to parental smoking and blood pressure/hypertension in children and adolescents. Second, blood pressure was measured using standard methods and strict quality control measures further ensured the quality of data [20].

There are also some limitations. First, the cross-sectional nature limited our ability of casual inference. Second, a proportion of participants were excluded due to incomplete information and our results may be subjective to selection bias. However, the included participants had a similar profile of demographic and anthropometric measures in comparison with the whole study population, indicating that the excluded participants were likely to be randomly distributed. Therefore, this exclusion should not seriously affect our results. Second, information on parental smoking was collected by questionnaire and no objective measurements such as blood/urinary cotinine levels were used. However, previous studies have shown a good agreement between self-reported smoking and biochemical markers both in adults and in children exposed to parental smoking [22, 23]. An additional limitation is that the questionnaire used in the present study was non-anonymous and this may have resulted in underreported active smoking rates. Consequently, it was possible that not all active smokers had been excluded and we were not able to completely rule out the influence of active smoking. However, in our subgroup analysis, we found similar results in the younger students, which were less likely to be affected by active smoking. Therefore, our overall findings on the adverse effects of parental smoking on blood pressure should not be seriously affected.

## Conclusions

In conclusion, in this large national sample of Chinese children and adolescents, exposure to parental smoking was associated with increased blood pressure and higher prevalence of hypertension in girls but not in boys. Our findings support the urgent needs to develop effective strategies to promote smoking-free environment, especially for children and adolescents.

## Additional files

Additional file 1: Characteristics of all participants and those included in data analysis. (DOCX 15 kb)

Additional file 2: Associations between exposure to parental smoking and blood pressure and hypertension in subgroup analysis. (DOCX 18 kb)

## Abbreviations

BMI: body mass index
CI: confidence interval
DBP: diastolic blood pressure
MS: metabolic syndrome
OR: odds ratio
SBP: systolic blood pressure
SD: standard deviation
