# Supplementary material for: Parental smoking and blood pressure in children and adolescents: a national cross-sectional study in China
Source: BMC Pediatr. 2019 Apr 18;19:116. doi: 10.1186/s12887-019-1505-8 (PMC6474055; doi:10.1186/s12887-019-1505-8)
Supplement: Supplementary file 1 — Characteristics of all participants and those included in data analysis. (DOCX 15 kb) [file 12887_2019_1505_MOESM1_ESM.docx]

| Additional file 1 Characteristics of all participants and those included in data analysis | | | | |
| --- | --- | --- | --- | --- |
| Characteristics |  | All (N=599, 59) |  | In-analysis (N=42, 745) |
| Age (year) |  | 11.5 (3.2) |  | 11.3 (3.1) |
| Sex (boys) |  | 30, 904 (51.5%) |  | 21, 463 (50.2%) |
| Weight (kg) |  | 41.6 (15.4) |  | 41.1 (16.3) |
| Height (cm) |  | 146.8 (16.6) |  | 145.9 (15.3) |
| Body mass index (kg/m^2^) |  | 18.6 (3.8) |  | 18.6 (3.8) |
| Systolic blood pressure (mmHg) |  | 104.7 (12.1) |  | 104.6 (12.1) |
| Diastolic blood pressure (mmHg) |  | 66.5 (8.8) |  | 66.3 (8.7) |

Descriptive statistics are presented as mean (SD) and number (percentage) for continuous variables and categorical variables, respectively.
