# Supplementary material for: Parental smoking and blood pressure in children and adolescents: a national cross-sectional study in China
Source: BMC Pediatr. 2019 Apr 18;19:116. doi: 10.1186/s12887-019-1505-8 (PMC6474055; doi:10.1186/s12887-019-1505-8)
Supplement: Supplementary file 2 — Associations between exposure to parental smoking and blood pressure and hypertension in subgroup analysis. (DOCX 18 kb) [file 12887_2019_1505_MOESM2_ESM.docx]

| Additional file 2 Associations between exposure to parental smoking and blood pressure and hypertension in subgroup analysis | | | | | | |
| --- | --- | --- | --- | --- | --- | --- |
| Blood Pressure |  | Boys | |  | Girls | |
|  |  | Coeff (95% CI) | *P* |  | Coeff (95% CI) | *P* |
| **SBP** (mmHg) |  |  |  |  |  |  |
| Younger (age: 7-12 years) |  | -0.13 (-0.44,0.19) | 0.43 |  | 0.40 (0.07,0.07) | 0.02 |
| Older (age:13-18 years) |  | 0.63 (0.07,1.19) | 0.03 |  | 0.26 (0.05,1.01) | 0.05 |
| **DBP** (mmHg) |  |  |  |  |  |  |
| Younger (age:7-12 years) |  | -0.08 (-0.33,0.17) | 0.51 |  | 0.22 (-0.04,0.48) | 0.10 |
| Older (age:13-18 years) |  | 0.30 (-0.12,0.72) | 0.16 |  | 0.34 (-0.03,0.71) | 0.07 |
| **Hypertension** (Odds ratio) |  |  |  |  |  |  |
| Younger (age:7-12 years) |  | 0.93 (0.85,1.01) | 0.10 |  | 1.16 (1.05,1.27) | 0.002 |
| Older (age:13-18 years) |  | 0.95 (0.78,1.15) | 0.58 |  | 0.96 (0.81,1.14) | 0.62 |

Participants reporting no exposure to parental smoking served as reference group.

Results were adjusted for height, body mass index, lifestyle factors (exercise, fruit intake and vegetable intake), maternal educational level and parental hypertension.

Abbreviations: CI, confidence interval; DBP, diastolic blood pressure; OR, odds ratio; SBP, systolic blood pressure.
